# Quantification of cerivastatin toxicity supports organismal performance assays as an effective tool during pharmaceutical safety assessment

**DOI:** 10.1111/eva.12365

**Published:** 2016-04-15

**Authors:** Shannon M. Gaukler, James S. Ruff, Tessa Galland, Tristan K. Underwood, Kirstie A. Kandaris, Nicole M. Liu, Linda C. Morrison, John M. Veranth, Wayne K. Potts

**Affiliations:** ^1^ Department of BiologyUniversity of UtahSalt Lake CityUTUSA; ^2^ Los Alamos National LaboratoryEnvironmental Stewardship GroupLos AlamosNMUSA; ^3^ Department of Pharmacology and ToxicologyUniversity of UtahSalt Lake CityUTUSA

**Keywords:** fitness assay, intraspecific competition, pharmaceutical development, reproduction, semi‐natural enclosures, toxicity assessment

## Abstract

A major problem in pharmaceutical development is that adverse effects remain undetected during preclinical and clinical trials, but are later revealed after market release when prescribed to many patients. We have developed a fitness assay known as the organismal performance assay (OPA), which evaluates individual performance by utilizing outbred wild mice (*Mus musculus*) that are assigned to an exposed or control group, which compete against each other for resources within semi‐natural enclosures. Performance measurements included reproductive success, survival, and male competitive ability. Our aim was to utilize cerivastatin (Baycol^®^, Bayer), a pharmaceutical with known adverse effects, as a positive control to assess OPAs as a potential tool for evaluating the safety of compounds during preclinical trials. Mice were exposed to cerivastatin (~4.5 mg/kg/day) into early adulthood. Exposure ceased and animals were released into semi‐natural enclosures. Within enclosures, cerivastatin‐exposed females had 25% fewer offspring and cerivastatin‐exposed males had 10% less body mass, occupied 63% fewer territories, sired 41% fewer offspring, and experienced a threefold increase in mortality when compared to controls. OPAs detected several cerivastatin‐induced adverse effects indicating that fitness assays, commonly used in ecology and evolutionary biology, could be useful as an additional tool in safety testing during pharmaceutical development.

## Introduction

A major problem during the pharmaceutical development process is that some adverse effects go undetected and are only discovered after a drug has been released onto the market and has become widely used. For example, 73% of pharmaceuticals that pass the preclinical phase of testing (conducted in nonhuman models to assess safety) fail during human clinical trials (Lipsky and Sharp 2001) and furthermore, 10% of all FDA (Food and Drug Administration) approved pharmaceuticals fail after market release due to unforeseen adversity (Schuster et al. 2005). Not only is drug development fraught with risk but it also requires between 12 and 15 years of research and typically costs companies $1.4 billion per drug (Miller 2012). One potential cause of numerous drugs failing after years of research is the inability of current preclinical methodologies to detect adverse drug reactions without ambiguous interpretation. Important organism‐level factors such as sensory perception, response to stimuli, and social behavior are controlled by multiple pathways. A major limitation of current drug‐safety evaluation is that assays for specific biomolecules in cell culture and in experimental animals can detect statistical differences, but provide little information on whether observed simultaneous changes in multiple pathways are indicative of potential pathological responses, are a beneficial acclimation to the presence of the drug, or are biologically neutral (Currie 2012).

Detecting adverse drug reactions during preclinical trials may be improved by incorporating concepts and techniques used in ecology and evolutionary biology into safety assessment. Ecologists utilize field studies to assess organisms in their natural context and often find that the consequences of treatments can be very different than under laboratory conditions, with numerous examples in areas as diverse as disease ecology (Pigeault et al. 2015), behavioral ecology (Calisi and Bentley 2009), and evolutionary genetics (Myers 1973). Additionally, evolutionary biologists often use fitness measures to determine the ultimate‐level consequence of competing alleles, or other manipulations, and they often arrive at different, more complete, conclusions than do those assessing more traditional proximate endpoints; these fitness assays have been used across a variety of model systems such as *Drosophila,* viruses and yeast [e.g., references (Shabalina et al. 1997; Thatcher et al. 1998; Lauring et al. 2012)]. By incorporating naturalistic environments into the study design adversities due to drug‐by‐environment interactions and those that involve cryptic effects across multiple physiological systems could be detected with higher efficacy because of the demanding nature of the environment brings to light a variety of outcomes, many of which will lead to decreased reproductive output, but some of which may be experimentally induced phenomena of interest that do not lead to decreased fitness.

We have developed a fitness assay, known as the organismal performance assay (OPA), which compares the fitness of exposed and control individuals that compete against each other under semi‐natural conditions. OPAs rely largely on wild behavioral phenotypes and as wild mice (*Mus musculus*) have co‐evolved with humans since the beginning of agriculture they exhibit natural behaviors in man‐made structures. The semi‐natural enclosures used in OPAs have comparable conditions (i.e., population density, light: dark cycle, access to resources and mates etc.) to wild environmental conditions that wild mice typically encounter; thus allowing for natural behaviors under this setting to be expressed. Outbred mice have more genetic polymorphisms than inbred strains, which allows for greater detection of adversity, as some adverse effects are caused by specific genotype‐by‐exposure interactions. OPAs utilize outbred wild‐derived mice that compete against each other for limited resources, such as food and mates, in a semi‐natural environment. Treatment and control individuals compete directly and the performance of individuals is measured with estimates of Darwinian fitness (i.e., lifetime reproductive success and survival) and the components leading to fitness (e.g., territoriality). The sensitivity of OPAs derives from the fact that outbred mice undergoing social competition reveal ultimate‐level defects in behavior or physiological performance that are otherwise cryptic as measureable negative outcomes, such as relegation to inferior habitat and reduced reproduction and survival. Consequently, reduced performance in almost any physiological system caused by a treatment should be detectable by the inability of exposed mice to perform at the high levels achieved by controls.

To assess the potential utility of OPAs in preclinical testing, two previous studies were conducted with pharmaceuticals that had passed both preclinical and clinical testing, but revealed adverse effects after public release, paroxetine (Paxil^®^) (Gaukler et al. 2015) and rofecoxib (Vioxx^®^) (Gaukler et al. 2016). Drugs with known adverse effects serve as a tool, or positive control, to support the ability of OPAs to evaluate pharmaceutical safety. Paroxetine is a selective serotonin reuptake inhibitor (SSRI), currently available on the market, and is suspected of causing birth defects in humans (Williams and Wooltorton 2005). When Gaukler et al. (2015) assessed the safety of paroxetine with OPAs, exposed females experienced an initial decline in reproduction and males experienced consistently reduced body mass, reproduction, and competitive ability when compared to controls. Rofecoxib, a nonsteroidal anti‐inflammatory drug (NSAID), was recalled in 2004 after being linked with an increased risk of cardiovascular events (Keane 2004). OPAs did not detect rofecoxib‐induced adverse effects, which could be explained by exposure design (i.e., small sample size), species differences, or that the benefits of the drug negated any side effects (Gaukler et al. 2016).

To further assess the utility of OPAs in pharmaceutical research, here we conduct a third study using cerivastatin (Baycol^®^, Lipobay^®^; Bayer, Leverkusen, Germany) as this drug was approved for market use in 1998, but was recalled in 2001 due to unacceptable health consequences (Wooltorton 2001). Specifically, cerivastatin was linked to 385 nonfatal and 52 fatal cases of rhabdomyolysis (the breakdown of skeletal muscle leading to kidney toxicity) in the 700 000 patients taking the drug (Furberg and Pitt 2001). Forty percent of fatalities were linked to a drug–drug interaction with gemfibrozil (Lopid^®^; Pfizer); however, when taken alone, cerivastatin was still 16–80 times more likely to cause rhabdomyolysis than other statins when prescribed at the highest therapeutic dose of 0.8 mg per day (Furberg and Pitt 2001; Pasternak et al. 2002). In addition to human cost in pain, suffering, and loss of life, cerivastatin also cost Bayer approximately $1.2 billion in settlement fees (Campbell et al. 2014).

Preclinical studies were conducted in several animal models to assess the effects of cerivastatin on teratogenicity, mutagenicity, carcinogenicity, and fertility (Keutz and Schluter 1998). Doses of 0.008–300 mg/kg/day were administered at either a single dose or in multiple doses over the duration of 4 weeks to 24 months. Adverse effects on the liver and muscle tissue were detected in rodent models, but cerivastatin was not teratogenic, mutagenic nor did it cause infertility or fecundity problems (Keutz and Schluter 1998). Preclinical trials suggested cerivastatin to have a similar toxicological profile to that of other statins whose adverse effects were primarily in muscle tissue and the liver (Keutz and Schluter 1998). These studies deemed cerivastatin safe, which supported moving cerivastatin into human clinical trials. Here, we use cerivastatin to further assess OPAs as a screening tool for adverse effects by determining whether cerivastatin exposure causes fitness declines in mice, which would indicate that OPAs may be developed into a useful tool that can add evolutionary fitness concepts to the endpoints studied by current food, drug, and chemical safety assessments.

## Materials and methods

### Animals

Outbred wild‐derived house mice were used in this experiment as they have retained behavioral traits that allow them to function successfully in semi‐natural environments, unlike mice from inbred lines (Nelson et al. 2013). Individuals used in this experiment were from the 12th generation of the colony described in Meagher et al. (2000), which were founded by 157 wild‐caught individuals captured near Gainesville, Florida. Regarding genetic diversity, our mice are likely more diverse than commercially available outbred mice that are often used in pharmacology; most of these mice have been in captivity for a century undergoing varying breeding regimens while under selection for breeding efficiency (Chia et al. 2005). Mice used in this study have lost genetic diversity since capture at an estimated rate of *H*_*t*_ = *H*_0_ (1−[1/2*N*_*e*_])^t^ where *H*_0_ is the initial heterozygosity and *H*_*t*_ is the heterozygosity after *t* generations after a decline of population size *N*_*e*_ (Kimura and Ohta 1969). Applying this logic, we estimate that our colony has lost only 12.8% of its initial heterozygosity as the smallest bottleneck was 44 successfully breeding animals. Furthermore, using a pedigree analysis, we calculated all pairwise coefficients of relatedness for a subset of 80 male mice during the 11th generation and the average relatedness coefficient was 0.0839, a value comparable to those reported between males in nature (Cunningham et al. 2013). Animals were kept on a 12:12 h light: dark cycle and provided with food and water *ad libitum*. All procedures and protocols were approved by IACUC at the University of Utah.

### Cerivastatin exposure

Mice were exposed to cerivastatin at approximately 4.5 mg/kg/day. Dosing was achieved by incorporating 1.5 g of cerivastatin (Sequoia Research Products, Pangbourne, United Kingdom; Molecular formula: C_26_H_33_FNNaO_5_) into 50 kg of standard rodent chow (TD.130006; Harlan Teklad, Madison, WI, USA), yielding a concentration equivalent to 30 mg of cerivastatin/kg of chow. As the concentration in the feed is known, a daily dose (or exposure) for a mouse can be calculated if the average daily food intake and the weight are known. As wild mice eat ~3 g per day, and assuming the average mouse weighs 20 g (Reagan‐Shaw et al. 2008), then individuals received ~0.09 mg of cerivastatin daily, or ~4.5 mg/kg/day. Using a metabolic rate conversion factor, this is equivalent to a human dose of ~0.36 mg/kg/day, or a daily dose of ~21.6 mg, assuming the average human weighs 60 kg (Reagan‐Shaw et al. 2008); cerivastatin was prescribed up to a daily dose of 0.8 mg (Furberg and Pitt 2001). Cerivastatin doses between 25 and 125 mg/kg of chow achieve serum levels in mice that fall within the range of human serum levels when taking therapeutic doses of cerivastatin (Shimizu et al. 2003). Only one dose was selected for this study and was chosen to mimic human therapeutic‐dose serum levels (Shimizu et al. 2003). Additionally, dose–response relationships were secondary to the goal of determining whether the OPA could detect adverse effects to a human‐relevant drug exposure. The dose used in this study was ~27 times higher than human therapeutic doses, but still within the range of doses (0.008–300 mg/kg) and exposure duration (4 to 24 months) used in preclinical testing (Keutz and Schluter 1998); therefore, the dose selected here is appropriate and consistent with other preclinical studies.

**Figure 1:**
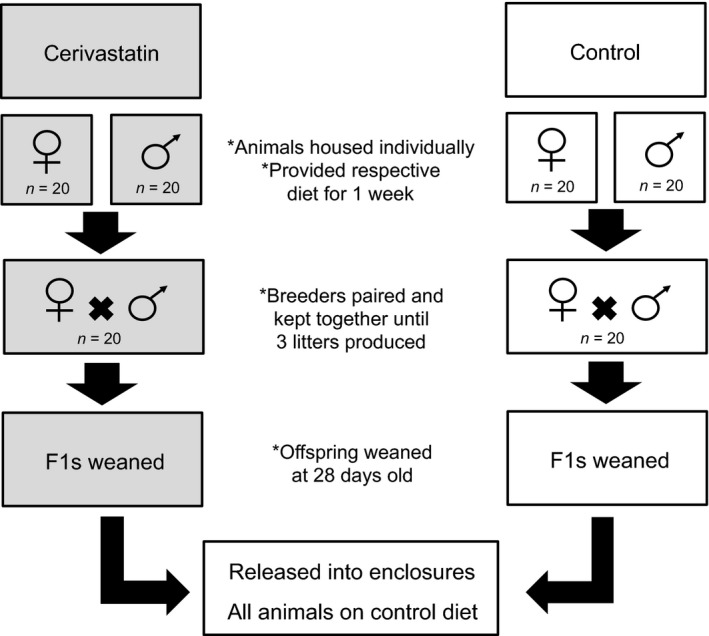
Schematic of cerivastatin exposure. Forty females and forty males were selected and individually housed; half of each sex were exposed to ~4.5 mg/kg/day of cerivastatin one week prior to pairing while the other half received the control diet. Breeders were paired and kept together until a maximum of three litters were produced; exposure to cerivastatin continued. Once litters were 28 days old, offspring were weaned and separated into cages with same‐sex siblings; exposure to cerivastatin continued. Upon early adulthood, males and females from both treatments were released into semi‐natural enclosures. Males were on average 15.9 ± 3.9 (M ± SD) weeks old and females were 16.7 ± 4.3 weeks old at the time of enclosure release. Once animals were in enclosures, cerivastatin exposure ceased and all animals were provided with the control diet. Gray shaded boxes depicts when cerivastatin exposure occurred.

Forty breeding pairs were randomly and equally assigned to either cerivastatin or control treatments. Females and males were kept separate for 1 week prior to breeding. Cerivastatin females were exposed 7 days prior to pairing and males were exposed 6 days prior. Breeders were kept together until they produced a maximum of three litters. At 28 days, pups were weaned and separated into same‐sex sibling cages. Data collected during weaning consisted of litter size, offspring mass, and sex. Exposure continued until pups became adults at which time they were released into enclosures with control animals [males and females were 15.9 ± 3.9 (M ± SD) and 16.7 ± 4.3 weeks old, respectively (Fig. 1)]. This duration provided substantial cerivastatin exposure to allow OPAs to detect health consequences. This is important because once animals were released into the semi‐natural enclosures, they were all fed the control diet because we are currently unable to keep animals on their respective diets while they are free‐ranging in enclosures. While control animals could have been switched to the cerivastatin treatment, switching the cerivastatin‐exposed animals to the control diet was a more conservative approach of detecting fitness declines, as any declines would represent persistent effects from a previous exposure.

### Semi‐natural enclosures

Enclosures have been extensively described in Ruff et al. (2013). Briefly, enclosures were ~30 m^2^ and contained four optimal and two suboptimal territories (Fig. S1). Optimal territories contained dark, enclosed nesting sites, whereas suboptimal nesting sites were exposed to light. Boundaries between territories were established with wire mesh fencing that was easily climbed, but provided spatial complexity. Each territory contained access to food and water and a 12:12 h light: dark cycle was maintained.

Five independent OPA experimental groups were established, each consisted of eight males and 12–16 females for a total of 116 (40 males, 76 females) individuals that are referred to as population founders. The space and animal density is consistent with the range of observations from the wild (Sage 1981). Half of the individuals of each sex were exposed to cerivastatin while the remaining half were control animals. Males were on average 15.9 ± 3.9 (M ± SD) weeks old and females were 16.7 ± 4.3 weeks old at the time of enclosure release. Control males were selected from litter sizes with an average of 4.7 ± 1.8 offspring per litter, and cerivastatin males were selected from litter sizes with an average of 4.0 ± 1.7 offspring per litter. Control females were selected from litter sizes with an average of 5.4 ± 1.9 offspring per litter, and cerivastatin females were selected from litter sizes with an average of 5.2 ± 1.5 offspring per litter. To prevent incidental breeding before territories were established, males were released into the enclosures with nonexperimental females. One week later, after territories were established, these nonexperimental females were removed and replaced with experimental females. Male founders were unrelated at the cousin level or above and each population consisted of one to three pairs of sisters (common in natural populations), but the relatedness between females was balanced between treatments. Populations were maintained for 28 weeks.

### Male competitive ability

One week prior to release, all animals (including founders of both sexes) received a passive integrated transponder (PIT) tag (TX1400ST; BioMark, Boise, ID, USA) for identification purposes. PIT tag antennas were placed on top of feeding stations within each of the six territories in an enclosure and data from PIT tag readers (FS2001F‐ISO; BioMark) were continuously downloaded to a computer containing data‐logging software (Minimon, Culver City, CA, USA). Two sets of PIT tag readers and antenna were used to collect data on animals and were rotated twice weekly among contemporaneous populations. Males were assigned as territorial occupants when an individual triggered >80% of all male PIT tag readings at a particular territory over a multiday reading frame. Female settlement data were also gathered via PIT tags, but are not reported here.

### Reproductive success

To quantify reproductive success, offspring were removed every 5 weeks, sacrificed, and had a tissue sample collected for genetic analysis. These ‘pup sweeps’ prevented the young from reaching sexual maturity, breeding with the founders and confounding the reproductive data. The first pup sweep occurred at week eight; the oldest offspring during this sweep were 5 weeks old as the gestation period is 3 weeks. A total of 1668 samples were collected with an average of 333.6 ± 105.3 (M ± SD) offspring per population.

In four of the five populations, reproductive success was determined on a population level by examining sex‐specific allelic variants described in Meagher et al. (2000). Briefly, in each population, founding individuals of each treatment were selected based on nonoverlapping allelic variants on the Y‐chromosome for males and on the mitochondrial genome for females. To control for confounding effects, such as segregating genes linked with the markers, marker assignment was balanced across treatments among populations. Mitochondrial genotypes were assessed in 1280 samples (four of five populations) and obtained for 100% of offspring. Y‐chromosome genotypes were assessed in all populations to determine reproductive success of males. Of the 1668 offspring, 818 Y‐chromosome genotypes were obtained, suggesting a sex ratio of 49:51 (M: F) and that 98% of all males were typed if the sex ratio was 1:1. Given that the sex ratio does not deviate from the expected 50:50, it is assumed that fathers sire as many daughters as they do sons; therefore, quantifying male reproductive success by only counting sons is likely to accurately reflect a proportion of total reproductive success. Female reproductive success was determined by parentage analysis using multiple microsatellite loci in the one population not typed with the above method to gain more knowledge on individual founder reproductive success for another study and is described in the supplementary information.

### Survivorship

Survivorship was assessed by daily noninvasive health checks and by extensive checks during pup sweeps. As part of the standard operating procedures for OPAs, the extensive checks were conducted every 5 weeks as to minimize disturbance and to not disrupt territoriality formation, which can increase infanticidal behavior (Parmigiani et al. 1990). Research personnel only entered the pens to rotate PIT‐tag readers, fill feeders, freshen waterers, and conduct pup sweeps. Dead founders were removed and identified by PIT tags. The date of death was estimated based upon the condition of the corpse. Animals that were dead long before discovery were given a death date halfway in between the last PIT tag read and the date the individual was found.

### Statistical analyses

Mass of offspring at weaning was analyzed with a linear mixed‐effects model (LMM), which assessed the fixed effects of exposure, litter order, and their interaction, while cage was modeled as a random effect with a random intercept generated for each. The model intercept was set to litter one for all breeding cage data sets. The sexes were analyzed separately to mirror models used to assess mass in OPAs and because body mass is a sexually dimorphic trait in mice (Dewsbury et al. 1980). There were 45 daughters from 11 cerivastatin cages, 79 daughters from 16 control cages for a total of 124 observations; 48 sons from 12 cerivastatin cages and 88 sons from 17 control cages for a total of 136 observations.

Litter size data (at weaning) are discrete counts and therefore were analyzed with a generalized linear mixed model (GLMM) with a Poisson distribution and logarithmic link. The model assessed the fixed effects of exposure, litter order and their interaction, while modeling cage as a random effect with a random intercept generated for each. There were 24 cerivastatin‐exposed litters from 12 cages and 44 control litters from 18 cages for a total of 68 observations.

Adult body mass was analyzed with a LMM which assessed the fixed effects of exposure, time, and their interaction on the 116 population founders. Population was modeled as a random effect with slopes and intercepts generated for each. Sexes were analyzed separately because the influence of pregnancy fundamentally alters changes in body mass over time and the intercept was set at week zero, as this was when founders were released. Measurement of founder mass continued for surviving individuals at each pup sweep. There were a total of 438 observations from 76 females and 196 observations from 40 males.

To assess the probability of territorial ownership, a GLMM with a binomial distribution and logit link was used. There were six territories within a population that were either occupied by cerivastatin‐exposed males, control males, or unoccupied. The model assessed the fixed effects of exposure, time, and their interaction, while population was set as a random effect with a random intercept generated for each. The model intercept was set to week three, as that was when data existed for each population and a total of 130 observations were collected.

Reproductive success in enclosures, in terms of total offspring, was analyzed with a GLMM with a Poisson distribution and logarithmic link. The model assessed the fixed effects of exposure, time, and their interaction, while population was set as a random effect with a random intercept calculated for each. The intercept was set at week eight, as that was when the first pup sweep occurred. Reproductive success for each treatment was measured five times over the course of the 28‐week study in each of the five independent populations for a total of 50 observations.

A multivariate Cox proportional hazard model, including exposure and population, analyzed the survival of founders. Individuals that survived the length of the study or that were intentionally removed from the study were censored. Female survival was not analyzed because of 100% survival in control females and only three mortalities in the cerivastatin exposure (76 individuals, three events). For male survival analyses, 40 individuals were analyzed, 15 events and 25 censorings.

Cox proportion hazard models were conducted in JMP 9.0.3 (SAS institute Inc., Cary NC). Both LMMs and GLMMs were conducted in R 3.0.2 using the lme4 library (Bates et al. 2014; R Development Core Team, 2013). *P*‐values were calculated for LMMs with the Swatterthwaite approximation under the lmerTest function (Kuznetsova et al. 2013). For all mixed models, several candidate models were fit to the data. These models varied in terms of random effects that estimated both intercept and/or slope. For each analysis, the model that had the lowest Akaike information criterion (AIC) score was reported. Neither the significance of a fixed effect nor the magnitude of the estimate varied between candidate models.

## Results

### Breeding cage measurements

There were no effects of cerivastatin exposure, litter order, or litter order by exposure on wean mass. No difference was detected in wean mass of cerivastatin‐exposed first litters when compared to controls (LMM; *n *=* *27, female offspring, *t *=* *0.68, *P *=* *0.51; *n *=* *29, male offspring, *t *=* *0.71, *P *=* *0.48). Cerivastatin‐exposed females were 11.18 ± 0.55 g (M ± SE), and controls were 10.81 ± 0.35 g. Cerivastatin‐exposed males were 12.36 ± 0.53 g, and controls were 11.98 ± 0.34 g. No effect of litter order (LMM; female, *t *=* *1.30, *P *=* *0.22; male, *t *=* *1.67, *P *=* *0.11) or litter order by exposure occurred (LMM; female, *t *= −0.81, *P *=* *0.43; male, *t *= −0.56, *P *=* *0.58). For a complete readout of mixed model outputs for breeding cage data (wean mass and litter size), see Table S1.

There was no effect of cerivastatin exposure, litter order, or litter order by exposure on litter size. Litter size was not affected by cerivastatin in first litters (GLMM; *n *=* *30, *z *= −0.04, *P *=* *0.97). Cerivastatin‐exposed breeders produced 3.51 (SE +0.83, −0.67) pups and control breeders produced 3.49 (+0.50, −0.44) pups in their first litter. No effect of litter order (GLMM; *z *=* *0.10, *P *=* *0.92) or litter order by exposure occurred (GLMM; *z *=* *1.00, *P *=* *0.32). Reported SEs are asymmetric as they were back transformed from logarithmic data that were generated in a GLMM.

### OPA measurements

**Figure 2:**
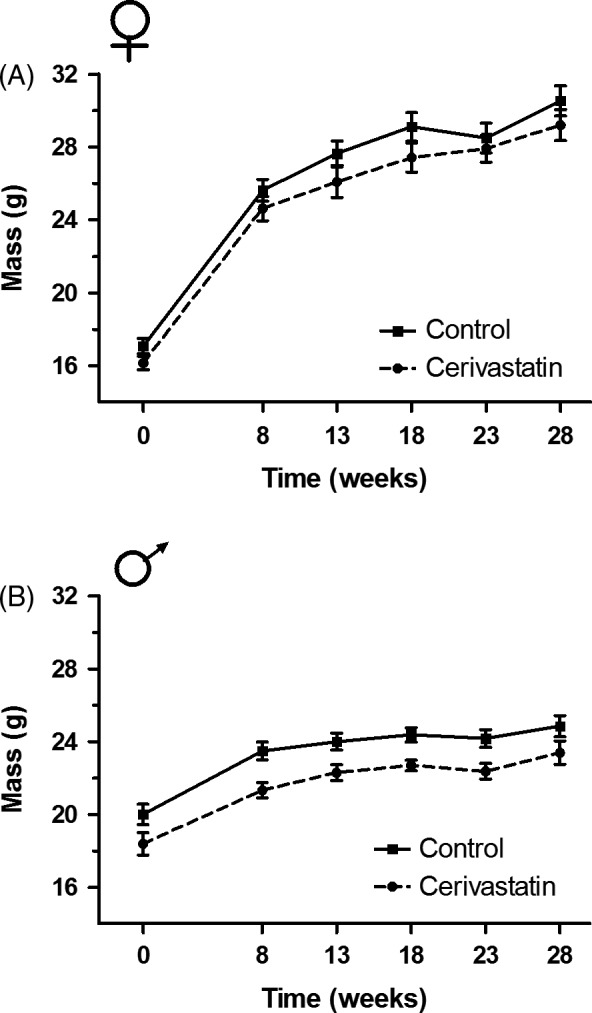
Body mass of cerivastatin‐exposed founders and controls over time in enclosures. (A) Cerivastatin‐exposed females had a 5% reduction in body mass relative to controls across the study which was marginally significant [*n *=* *76 mice, *observations* ^^= 483 (LMM;* t *= −1.74, *P *=* *0.09). (B) Cerivastatin‐exposed males had significantly less (10%) body mass than controls throughout the study [*n *=* *40 mice, *observations* ^^= 196 (LMM;* t *= −3.66, *P *<* *0.001). Time point 0 is when animals were released into enclosures. Lines connect means and error bars represent standard error.

Female founder mass was affected by cerivastatin exposure and time, but not by the interaction of time by exposure. A marginally significant trend was detected in which cerivastatin‐exposed females weighed 5% less than control females at week zero (LMM; *n *=* *76, *t *= −1.74, *P *=* *0.09; Fig. 2A). Cerivastatin‐exposed females were 18.69 ± 0.69 g (M ± SE), whereas control females were 19.89 ± 0.78 g. Females from both treatments gained mass over time, presumably due to pregnancy (LMM; *t *=* *10.14, *P *<* *0.001), but no interaction between time and exposure was detected (LMM; *t *=* *0.22, *P *=* *0.83), suggesting that the trend in reduced mass experienced by cerivastatin‐exposed females at the intercept continued throughout the study. For a complete readout of mixed model outputs for enclosure body mass data, see Table S2.

Male founder mass was affected by cerivastatin exposure and time, but not by the interaction of time by exposure. Cerivastatin‐exposed males had 10% less body mass than controls at week zero (LMM; *n *=* *40, *t *= −3.66, *P *<* *0.001; Fig. 2B). Cerivastatin‐exposed males were 19.30 ± 0.56 g (M ± SE), while control males were 21.35 ± 0.56 g. Males gained weight over time (LMM; *t *=* *9.53, *P *<* *0.001); however, no time by exposure interaction was detected (LMM; *t *=* *1.05, *P *=* *0.30), indicating that cerivastatin‐exposed males had less mass than controls throughout the study.

**Figure 3:**
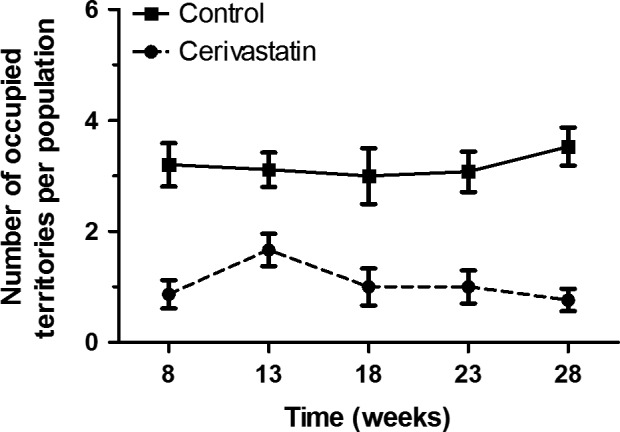
Competitive ability of cerivastatin‐exposed males and controls. Cerivastatin‐exposed males occupied 63% fewer territories than control males throughout the duration of the experiment [*n *=* *5 populations, *observations* ^^= 130 (GLMM;* z *= −4.55, *P *<* *0.001)]. Within each enclosure, there were six available territories and a male was considered a territorial occupant when an individual triggered >80% of all male PIT tag readings at a particular territory over a multiday reading frame. On average, control males occupied 49% of the territories, cerivastatin‐exposed males occupied 18% territories, and the remaining 32% territories were left undefended. The undefended territories still contain male mice however, <80% of male reads at this location belonged to a single individual. Points represent the mean number of territories of five populations. To aid in visualization, time points from 5‐week intervals have been pooled, except for the first time point consists of 8 weeks. For example, time point week eight consists of all data points collected from weeks 1–8; time points displayed at week 13 consists of all data points collected from weeks 9–13 and so on. Lines connect means of the five populations and error bars represent standard error.

Male competitive ability was negatively affected by cerivastatin exposure, but no effect of time or time by exposure was observed. Control males were 2.5 times more likely to occupy a territory than cerivastatin‐exposed males at week three. Control males occupied 49% of the territories, while cerivastatin‐exposed males only occupied 18% territories, leaving 32% territories undefended (GLMM; *n *=* *5, *z *= −4.55, *P *<* *0.001; Fig. 3). The percent of undefended territories is not unusual because 2/6 (or 33%) of the territories are suboptimal and are often difficult to defend. No effect of time (GLMM; *z *=* *0.99, *P *=* *0.32) or time by exposure were observed (GLMM; *z *= −1.12, *P *=* *0.26), indicating that cerivastatin‐exposed males acquired fewer territories over the duration of the study. For a complete readout of mixed model outputs for competitive ability, see Table S3.

**Figure 4:**
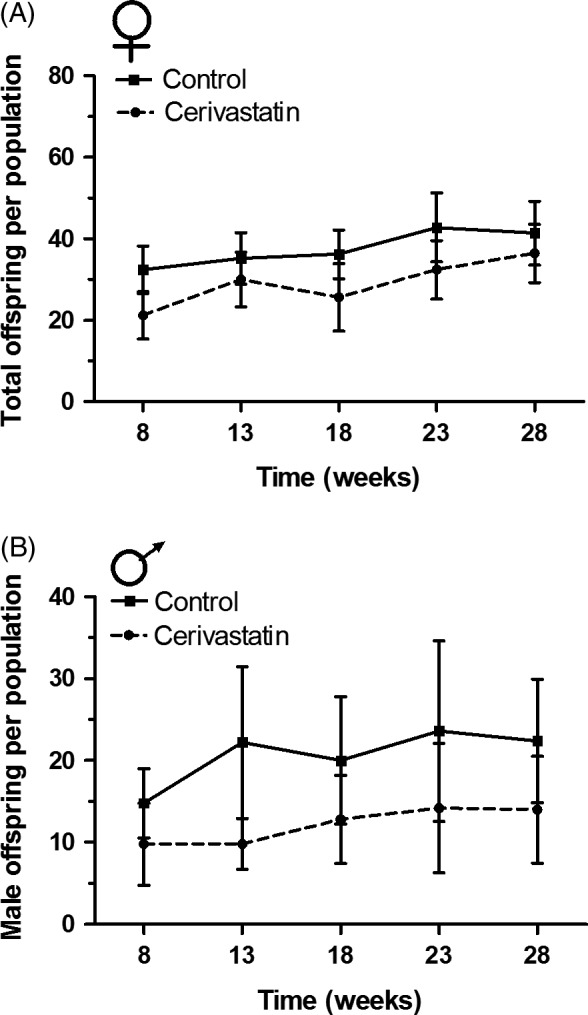
Reproductive success of cerivastatin‐exposed and control animals in enclosures. (A) Cerivastatin‐exposed females had 25% fewer offspring than controls throughout the study [*n *=* *5 populations, *observations* ^^= 50 (GLMM;* z *= −3.70, *P *<* *0.001)]. (B) Similarly, cerivastatin‐exposed males sired 41% fewer offspring than controls [*n *=* *5 populations, *observations* ^^= 50 (GLMM;* z *= −4.37, *P *<* *0.001)]. Female reproduction is in terms of total offspring, as mitochondrial markers and multiple microsatellite markers were used. Male reproduction is in terms of male offspring, as Y‐chromosomal markers were used. Lines connect means of the five populations at each time point for each sex and error bars represent standard error.

Female reproduction was affected by both cerivastatin exposure and time, but not by the interaction of time by exposure. Cerivastatin females experienced a 25% reduction in reproductive success when compared with controls (GLMM; *n *=* *5, *z *= −3.70, *P *<* *0.001; Fig. 4A). Cerivastatin‐exposed females had 21.82 (SE +2.09, −1.91) offspring per population, while controls had 30.63 (+5.40, −4.58) per population at week eight. Females from both treatments had more offspring over time (GLMM; *z *=* *3.39, *P *<* *0.001), but no time by exposure effect was detected (GLMM; *z *=* *0.89, *P *=* *0.38), suggesting the reproductive deficiency of cerivastatin‐exposed females at the intercept was consistent across the study. For a complete readout of mixed model outputs for reproduction in enclosures, see Table S4.

Male reproduction was affected by both cerivastatin exposure and time, but not by the interaction of time by exposure. Cerivastatin‐exposed males had 41% fewer male offspring than control males (GLMM; *n *=* *5, *z *= −4.37, *P *<* *0.001; Fig. 4B). Cerivastatin‐exposed males sired 9.14 (SE +1.31, −1.15) male offspring per population, while controls sired 16.42 (+3.29, −2.76) male offspring per population, at week eight. Males from both treatments had more offspring over time (GLMM; *z *=* *2.58, *P *<* *0.01), but no effect of time by exposure was detected (GLMM; *z *=* *0.49, *P *=* *0.62), indicating that the reproductive deficiency experienced by cerivastatin‐exposed males was consistent throughout the study.

**Figure 5:**
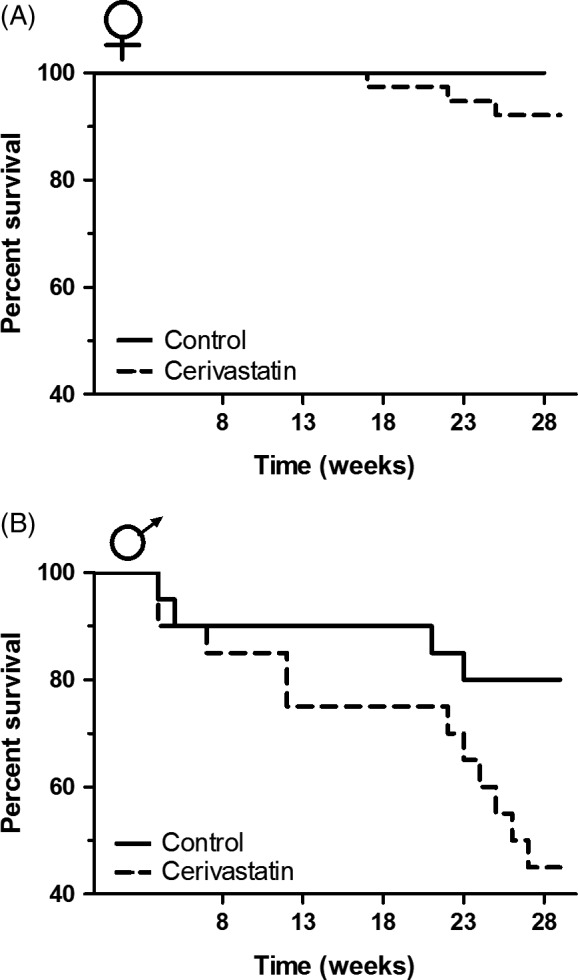
Survivorship of cerivastatin‐exposed animals and control animals in enclosures. (A) Cerivastatin‐exposed females (*n *=* *76) experienced reduced survival compared to controls; however, these data were not analyzed due to so few deaths (100% survival of control females and only three cerivastatin‐exposed mortalities). (B) Cerivastatin‐exposed males (*n *=* *40) experienced a threefold increase in mortality compared to their control counterparts (PH;* χ* ^2 ^= 4.79, *P *=* *0.03).

Cerivastatin‐exposed individuals experienced higher mortality than controls. Cerivastatin‐exposed females had increased mortality (*n *=* *76); however, the data were not analyzed due to overall low mortality; only three cerivastatin females died while 100% of control females survived (Fig. 5A). Cerivastatin‐exposed males experienced a mortality rate 3.0 times that of controls (PH; *n *=* *40, *χ* ^2^ = 4.79, *P *=* *0.03; Fig. 5B). Mortality rate did not differ across replicate populations (PH; *χ* ^2 ^= 1.65, *P *=* *0.80).

## Discussion

OPAs revealed numerous cerivastatin‐induced adverse phenotypes, which ultimately resulted in reduced health, performance, and reproductive success. Cerivastatin‐exposed males experienced a 10% reduction in body mass, occupied 63% fewer territories, sired 41% fewer offspring, and had a threefold increase in mortality compared to controls. Cerivastatin‐exposed females experienced a 5% marginally significant trend in reduced body mass and had significantly fewer (25%) offspring when compared to controls. Cerivastatin exposure likely disrupted several physiological mechanisms. These effects were observed when mice were exposed to cerivastatin that was ~27 times higher than human therapeutic doses, but mimicked human serum levels. In typical preclinical trials, a range of doses are assessed, which are used to inform doses in which human testing should begin (often 10–100 fold less than preclinical trials). However, as this study was retrospective, only one dose was assessed and it is unknown whether lower doses cause similar toxicological effects or whether these effects would be detected under other experimental contexts (i.e., lack of developmental exposure).

Cerivastatin‐exposed males experienced a reduction in body mass in enclosures, which may be attributable to muscle toxicity and degeneration. Statins in general are known to cause myotoxicity, but typically occur at a low rate while only 1–7% of patients taking statins experience this side effect (Sirvent et al. 2008). Other research has revealed that exercise can exacerbate statin‐induced myotoxicity (Sinzinger and O'Grady 2004). Unlike a caged environment, mice inhabiting semi‐natural enclosures engage in regular physical activity and males compete with each other to obtain and defend territories. It is possible that the increase in physical activity, made possible by the inclusion of ecologically relevant environment, caused myotoxicity in cerivastatin‐exposed male mice. The less severe weight differences in females may be due to less physical activity, while devoting more energy into reproductive efforts (Gittleman and Thompson 1988).

Cerivastatin‐exposed males were less competitive and dominated fewer territories than controls. The fact that there was no significant effect of time or of a time by exposure interaction indicates that cerivastatin‐exposed males failed to acquire territories at the same rate as controls; if differential rates of territory loss were responsible then a time by drug interaction would have been observed. Reduced competitive ability may be due to reduced body weight as males engage in physical competitions to obtain and defend territories. Larger body size has been shown to be beneficial in territorial acquisition and defense in mice (van Zegeren 1980; Krackow 1993). Alternatively, cerivastatin‐exposed males may have experienced muscle fatigue that hindered their endurance. Regardless of the mechanistic underpinnings cerivastatin‐exposed males were less capable of implementing social behaviors of keen importance to individual fitness (Carroll et al. 2004).

Both cerivastatin‐exposed males and females suffered reproductive declines when compared to controls. In a previous OPA study, dominant males were found to sire >80% of offspring born in enclosures (Carroll et al. 2004); therefore, the differences detected in competitive ability are likely to explain, at least in part, the differences detected in reproduction. As cerivastatin‐exposed males experienced a threefold increase in mortality, fewer males were reproducing and contributing to overall treatment‐level reproduction. The proximate mechanisms of reduced reproductive success in cerivastatin‐exposed females are unknown; however, it is possible that reduced cholesterol levels caused by cerivastatin exposure may have contributed to these findings. While OPAs detected adverse reproductive effects, these effects escaped the detection by traditional preclinical rodent assays (Keutz and Schluter 1998); this discrepancy again highlights the importance of including natural, or at least semi‐natural, stressors when assessing individual fitness measures.

**Figure 6:**
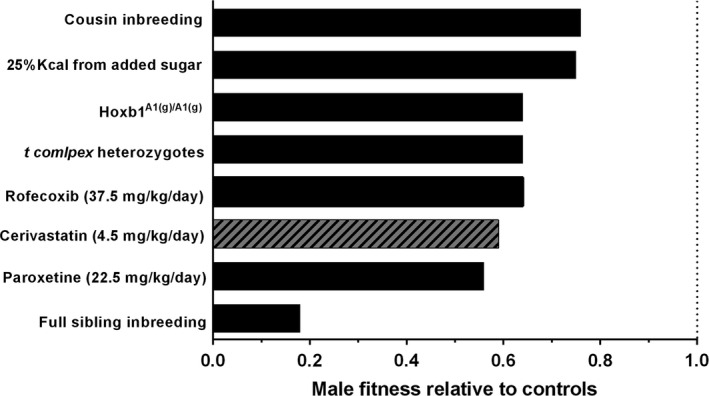
Comparisons of male fitness relative to control counterpart from published OPA experiments. Cerivastatin exposure had greater consequences on male fitness (reproductive success as determined via male offspring) when compared with cousin inbreeding, moderate sugar consumption and harboring a selfish gene, experienced comparable fitness declines to those males exposed to paroxetine, but suffered less fitness consequences when compared to inbreeding at the sibling level (Meagher et al. 2000; Carroll et al. 2004; Ilmonen et al. 2008; Ruff et al. 2013, 2015; Gaukler et al. 2015, 2016). Figure adapted from Ruff et al. (2013) and Gaukler et al. (2015).

In the case of cerivastatin, exposure caused more adversity to male fitness than female fitness. OPAs may have detected male adversity with greater sensitivity than females due to the competitive nature of males in a polygynous mating system, a phenomenon appreciated in evolutionary biology since Bateman (1948). However, OPAs had adequate sensitivity to detect adversity on female fitness due to cerivastatin exposure. This is not the first OPA study to detect differential health consequences between sexes as in four previous OPA studies, males have suffered greater negative fitness impacts than females (i.e., sired fewer offspring); these studies include inbreeding at the sibling level (Meagher et al. 2000), inbreeding at the cousin level (Ilmonen et al. 2008), when fed a moderate sugar diet (Ruff et al. 2013), and when exposed to the pharmaceutical paroxetine (Gaukler et al. 2015). As OPA studies generate the same outputs, relative fitness can be compared among treatments. Results from this study indicate that cerivastatin‐exposed male fitness (i.e., reproductive success) is approximately 25% less than the male progeny of parents that were first cousins (Fig. 6). Thus, it may be inferred that cerivastatin exposure causes substantially greater adverse effects than cousin‐level inbreeding, yet these health declines went undetected using more traditional preclinical methodologies.

Unlike proximate‐level based approaches of toxicity assessment, OPAs allowed for quantification of adverse impacts at the organismal level, requiring no assumptions of mechanistic cause. While many preclinical tests aim to reveal biochemical alterations or mechanistic changes, some nonmechanistic approaches are widely accepted, such as the functional observational battery (FOB), consists of behavioral assays and autonomic tests (Moser 2011) and the lesser used LD50 assay. The inability of OPAs to reveal mechanistic changes does not negate its value for supplementing current methods, but rather OPAs could provide an early warning of potential problems and for comparing the relative risk for alternative drug candidates. For example, an OPA study that reveals one candidate compound to cause fitness losses at ten times therapeutic levels while an alternative causes the same fitness losses at two time therapeutic levels would be valuable information for research prioritization even without knowing the biochemical mechanism. Additionally, many known toxic compounds lack definitive mechanistic understanding and during drug development, indication of toxicity is a reason to consider alternatives. Many proximate assays observe small, but statistically significant changes in tissue concentrations of specific biomolecules, but the physiological meaning of these treatment effects is unclear, especially when the measurements fall within the normal range for the species. In contrast, it is likely that any change in the OPA endpoints of survival and reproductive success has unambiguous meaning; health and performance have been degraded by the treatment and detecting these adverse phenotypes can help reveal the underlying mechanisms.

Pharmaceuticals are becoming more widespread in the environmental and most are designed to have biological effects at low levels; the examination of pharmaceutical‐induced effects on nontarget organisms is a relatively new field where environmental exposures have only been considered in the last 10–15 years (Arnold et al. 2014). Many medications are intended to cause alterations in behavior, physiology and reproduction, such as antidepressants and birth control, and can have analogous effects on nontarget species (Arnold et al. 2014). Pharmaceuticals have been demonstrated to pose risks to the individual, population, and community levels by both experimental and ecotoxicological derived studies; such studies have revealed alterations of behavior, physiology, reproduction, and mortality in response to pharmaceutical exposure (Oaks et al. 2004; Tyler and Jobling 2008; Bean et al. 2014; Brodin et al. 2014). Even if pharmaceuticals do not cause mortality or reproductive effects directly, other ecologically relevant endpoints such as predator avoidance behavior, efficient ability to forage, or intrasexual behaviors could be altered due to exposure that will reduce an organism's fitness (Perreault et al. 2003; Painter et al. 2009; Bean et al. 2014; Brodin et al. 2014). These endpoints are important to assess, but are often overlooked in traditional ecological risk assessments (Arnold et al. 2014); however, as OPAs encompass more ecologically relevant endpoints than traditional assays, OPAs may be able to be used to predict evolutionary responses of natural populations to these manmade chemicals.

OPAs have now been used to assess the safety of three pharmaceuticals with known adverse effects, paroxetine (Gaukler et al. 2015), rofecoxib (Gaukler et al. 2016), and cerivastatin. OPAs detected adversity in both paroxetine and cerivastatin, but did not detect rofecoxib‐induced adversity. Similar to current preclinical methodologies, OPAs are not expected to reveal all maladies. However, the fact that OPAs revealed health declines missed by conventional safety testing, suggests that OPAs could be a useful tool if added to current preclinical assessment methodologies. Additional research is warranted to assess OPAs utility in pharmaceutical safety research, such as assessing a wide range of doses and exposure durations, testing similar compounds (e.g., statins currently on the market) and testing pharmaceuticals known to be safe. In future OPA studies, the use of outbred wild‐derived mice and mimicking natural conditions such as population density, proportion of related individuals, sex ratios, light cycle, etc. is paramount. Future studies could also examine individual fitness rather than treatment‐level fitness and these data could be used to assess female dominance behaviors. Additionally, if the exposure occurred prior to the enclosure testing phase, continuing the exposure during the duration of the enclosure phase would be optimal; this could be possible with PIT‐tag feeders which would allow mice to continue on their respective diets. The major caveats in utilizing the OPA approach are the number of animals used as well as the duration of the experiments; while this is a cost in OPA assessment, the benefit of detecting adversity could outweigh the costs in many scenarios.

The success of OPAs ability to detect adversity stems from the ecological and evolutionary concepts that have been incorporated into the experimental design. The naturalistic environment and quantification of fitness measures as endpoints allows adverse effects from a treatment‐by‐environment to be detected with higher efficacy. Additionally, OPAs utilize outbred mouse strains, which have genetic polymorphisms that interact with treatment, thus increasing the likelihood that adverse effects will be detected in at least a subset of individuals. Fitness assays utilizing evolutionary concepts and techniques have revealed cryptic health consequences that would otherwise be unseen across several fields of research and it has now been demonstrated that these methodologies are functional in toxicity assessment.

## Data archiving statement

All raw data are available in ‘Cerivastatin Data S2’.

## Supporting information


**Figure S1.** An image of a semi‐natural enclosure used in OPA experiments.
**Table S1**. Mixed model outputs for litter size and wean mass.
**Table S2**. Linear mixed model outputs for founder body mass over time in enclosures.
**Table S3**. Generalized linear mixed model outputs for male competitive ability over time.
**Table S4**. Generalized linear mixed model outputs for reproduction over time.
**Data S1.** Reproductive success methods and statistical tables.

**Data S2.** Experimental data.
