# Supplementary material for: Quantification of cerivastatin toxicity supports organismal performance assays as an effective tool during pharmaceutical safety assessment
Source: Evol Appl. 2016 Apr 15;9(5):685–96. doi: 10.1111/eva.12365 (PMC4869410; doi:10.1111/eva.12365)
Supplement: Supplementary file 1 — Figure S1. An image of a semi‐natural enclosure used in OPA experiments. Table S1. Mixed model outputs for litter size and wean mass. Table S2. Linear mixed model outputs for founder body mass over time in enclosures. Table S3. Generalized linear mixed model outputs for male competitive ability over time. Table S4. Generalized linear mixed model outputs for reproduction over time. Data S1. Reproductive success methods and statistical tables. [file EVA-9-685-s001.doc]

**Supplementary information**

**
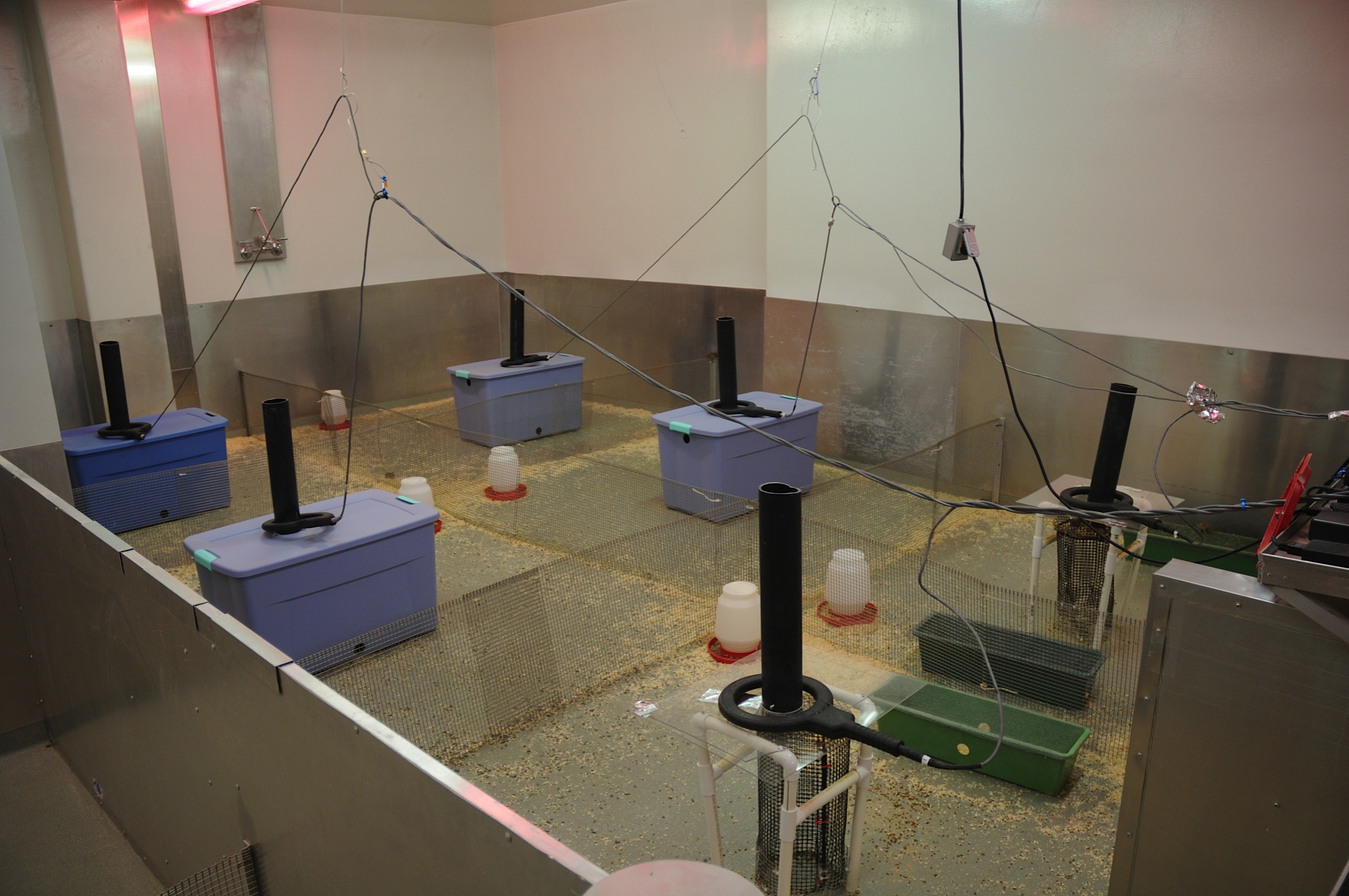
**

**Figure S1.** **An** **image of a semi-natural enclosure used in OPA experiments.** Each enclosure is ~30 m^2^ and contains six territories that are divided by wire mesh. The four optimal territories have the large blue bins, which contain multiple dark nesting sites and are defendable. The two suboptimal territories consist of light exposed nesting sites. Each territory contains food within the chimney-like structures and water (poultry waterers). PIT tag antennas (tennis racket like structures) are placed above each feeding site. Photograph courtesy Benjamin Sutter.

**Table 1**. Mixed model outputs for litter size and wean mass.

| **Female Wean Mass** | | LMM (groups = 27; observations = 124) | | | | | | |
| --- | --- | --- | --- | --- | --- | --- | --- | --- |
| *Random effects* | | *Variance* | | *Std. Dev.* | |  | | |
| Cage (Intercept) | | 1.3423 | | 1.1586 | |
| Cage (Slope) | | 0.2616 | | 0.5115 | |
| *Fixed effects* | | *Estimate* | | *Std. Error* | | *t value* | | *Pr( >｜t｜)* |
| Intercept (Litter 1) | | 10.8131 | | 0.3517 | | 30.7420 | | <0.0001*** |
| Exposure (Cerivastatin) | | 0.3676 | | 0.5445 | | 0.6750 | | 0.5060 |
| Litter Order | | 0.2769 | | 0.2134 | | 1.2980 | | 0.2180 |
| Exposure (Cerivastatin)×Litter Order | | −0.2998 | | 0.3725 | | −0.8050 | | 0.4300 |
| **Male Wean Mass** | | LMM (groups = 29; observations = 136) | | | | | | |
| *Random effects* | | *Variance* | | *Std. Dev.* | |  | | |
| Cage (Intercept) | | 0.9751 | | 0.9875 | |
| Cage (Slope) | | 0.3131 | | 0.5596 | |
| *Fixed effects* | | *Estimate* | | *Std. Error* | | *t value* | | *Pr( >｜t｜)* |
| Intercept (Litter 1) | 11.9781 | | 0.3381 | | 35.4200 | | <0.001*** | |
| Exposure (Cerivastatin) | 0.3805 | | 0.5378 | | 0.7100 | | 0.4840 | |
| Litter Order | 0.4470 | | 0.2675 | | 1.6700 | | 0.1110 | |
| Exposure (Cerivastatin)×Litter Order | −0.2711 | | 0.4817 | | −0.5600 | | 0.5790 | |
| **Litter Size** | GLMM with Poisson distribution and logarithmic link (groups = 30; observations = 68) | | | | | | | |
| *Random effects* | *Variance* | | *Std. Dev.* | |  | | | |
| Cage (Intercept) | 0.0798 | | 0.2824 | |
| *Fixed effects* | *Estimate* | | *Std. Error* | | *z value* | | *Pr( >｜z｜)* | |
| Intercept (Litter 1) | 1.2491 | | 0.1335 | | 9.3550 | | <0.001*** | |
| Exposure (Cerivastatin) | 0.0074 | | 0.2124 | | 0.0350 | | 0.9720 | |
| Litter Order | 0.0097 | | 0.0988 | | 0.0980 | | 0.9220 | |
| Exposure (Cerivastatin)×Litter Order | 0.1771 | | 0.1763 | | 1.0040 | | 0.3150 | |

*** Indicates a *P* value < 0.001.

**Table 2**. Linear mixed model outputs for founder body mass over time in enclosures.

| **Female Body Mass** | LMM (group = 76; observations = 438) | | | | | |
| --- | --- | --- | --- | --- | --- | --- |
| *Random effects* | *Variance* | | *Std. Dev.* | |  | |
| Individual (Intercept) | 0.4176 | | 0.6462 | |
| Individual (Slope) | 0.0209 | | 0.1447 | |
| Population (Intercept) | 1.8593 | | 1.3636 | |
| Population (Slope) | 0.0024 | | 0.0489 | |
| *Fixed effects* | *Estimate* | | *Std. Error* | | *t value* | *Pr( >｜t｜)* |
| Intercept (Week 0) | 19.8851 | | 0.7814 | | 25.4490 | <0.001*** |
| Exposure (Cerivastatin) | −1.1998 | | 0.6917 | | −1.7350 | 0.0857 ∙ |
| Time | 0.4309 | | 0.0425 | | 10.1400 | <0.001*** |
| Exposure (Cerivastatin)×Time | 0.0114 | | 0.0519 | | 0.2200 | 0.8263 |
| **Male Body Mass** | LMM (group = 40; observations = 196) | | | | | |
| *Random effects* | *Variance* | *Std. Dev.* | |  | | |
| Individual (Intercept) | 1.7187 | 1.3110 | |
| Population (Intercept) | 0.8147 | 0.9026 | |
| *Fixed effects* | *Estimate* | *Std. Error* | | *t value* | | *Pr( >｜t｜)* |
| Intercept (Week 0) | 21.3518 | 0.5636 | | 37.8900 | | <0.001*** |
| Exposure (Cerivastatin) | −2.0497 | 0.5605 | | −3.6600 | | <0.001*** |
| Time | 0.1482 | 0.0156 | | 9.5300 | | <0.001*** |
| Exposure (Cerivastatin)×Time | 0.0246 | 0.0235 | | 1.0500 | | 0.2970 |

∙ Indicates a *P* value <0.10, *** < 0.001.

**Table 3**. Generalized linear mixed model outputs for male competitive ability over time.

| **Male Competitive Ability** | GLMM with binomial distribution and logit link (group = 5; observations = 130) | | | |
| --- | --- | --- | --- | --- |
| *Random effects* | *Variance* | *Std. Dev.* |  | |
| Population (Slope) | 0.0570 | 0.2387 |
| *Fixed effects* | *Estimate* | *Std. Error* | *z value* | *Pr( >｜z｜)* |
| Intercept (Week 3) | −0.0260 | 0.2237 | −0.1160 | 0.9080 |
| Exposure (Cerivastatin) | −1.4625 | 0.3215 | −4.5490 | <0.001*** |
| Time | 0.0125 | 0.0126 | 0.9890 | 0.3230 |
| Exposure (Cerivastatin)×Time | −0.0235 | 0.0209 | −1.1230 | 0.2620 |

*** Indicates a *P* value < 0.001.

**Table 4**. Generalized linear mixed model outputs for reproduction over time.

| **Female Reproduction** | | GLMM with Poisson distribution and logarithmic link (group = 5; observations = 50) | | | |
| --- | --- | --- | --- | --- | --- |
| *Random effects* | *Variance* | | *Std. Dev.* |  | |
| Population (Intercept) | 0.11328 | | 0.3366 |
| *Fixed effects* | *Estimate* | | *Std. Error* | *z value* | *Pr( >｜z｜)* |
| Intercept (Week 8) | 3.4221 | | 0.1622 | 21.1020 | <0.001*** |
| Exposure (Cerivastatin) | −0.3394 | | 0.0917 | −3.7000 | <0.001*** |
| Time | 0.0163 | | 0.0048 | 3.3910 | <0.001*** |
| Exposure (Cerivastatin)×Time | 0.0063 | | 0.0071 | 0.8850 | 0.3764 |
| **Male Reproduction** | GLMM with Poisson distribution and logarithmic link (group = 5; observations = 50) | | | | |
| *Random effects* | *Variance* | | *Std. Dev.* |  | |
| Population (Intercept) | 0.1345 | | 0.3668 |
| *Fixed effects* | *Estimate* | | *Std. Error* | *z value* | *Pr( >｜z｜)* |
| Intercept (Week 8) | 2.7980 | | 0.1832 | 15.2690 | <0.001*** |
| Exposure (Cerivastatin) | −0.5858 | | 0.1341 | −4.3660 | <0.001*** |
| Time | 0.0162 | | 0.0063 | 2.5820 | 0.0098** |
| Exposure (Cerivastatin)×Time | 0.0051 | | 0.0103 | 0.4910 | 0.6233 |

** Indicates a *P* value < 0.01, *** < 0.001.

**Reproductive success**

In one of five populations, female reproductive success was determined by parentage analysis using multiple microsatellite loci to gain more knowledge on individual founder reproductive success for another study. These data were converted to treatment-level readouts (i.e., number of pups per treatment rather than number of pups per individual) and combined with the mitochondrial data for analysis. Between five and 11 autosomal microsatellite loci were amplified, scored and analyzed in a stepwise fashion. Loci used were: d1mit449, d3mit22, d3mit312, d3mit333, d5mit139 d6mit138, d9mit251, d12mit277, d14mit128, d17mit62 and d19mit110. Primer sequences were obtained from the Mouse Genome Informatics website, The Jackson Laboratory, Bar Harbor Maine (The Jackson Laboratory 2013). Primers were fluorescently tagged with either CY-5 or CY-3 dye. PCR products were run on a 14” x 17”, 6.25% denaturing acrylamide gel at 40 W for three to seven hours (locus dependent). Gels containing tagged DNA fragments were imaged on a Typhoon Scanner 8600 with ImageQuant software (Amersham Biosciences, Piscataway, NJ).

Parentage was assigned by using Cervus 3.0 (Kalinowski, Taper, and Marshall 2007). Allele frequencies were calculated using the genotypes of all candidate mothers and fathers and all offspring within the population. Simulations were run at 10,000 cycles with an error rate of 1%. Assigned parents were accepted when the trio confidence of mother, father and offspring was 95%. With this rule, 97% offspring (375 of 388) were assigned to parent pairs.

**Literature Cited**

Kalinowski, S., M. Taper, and T. Marshall. 2007. Revising How the Computer Program CERVUS Accommodates Genotyping Error Increases Success in Paternity Assignment. Molecular Ecology **16**: 1099–1106.

The Jackson Laboratory. 2013. http://www.informatics.jax.org.
